# The effect of dietary factors and nutrients on osseointegration, dental implant success and survival: a scoping review

**DOI:** 10.1186/s40729-026-00680-8

**Published:** 2026-04-06

**Authors:** Buyanbileg Sodnom-Ish, Sebastian Kühl, Valentin Herber, Michael M. Bornstein

**Affiliations:** 1 Department of Oral Health & Medicine, University Center for Dental Medicine Basel UZB, University of Basel, Mattenstrasse 40, Basel, 4058 Switzerland; 2 Department of Periodontics and Endodontics, School of Dentistry, Mongolian National University of Medical Sciences, Ulaanbaatar, Mongolia; 3 Department of Oral Surgery, University Center for Dental Medicine Basel UZB, University of Basel, Mattenstrasse 40, Basel, 4058 Switzerland

**Keywords:** Dietary supplements, Dental implants, Osseointegration, Vitamin D, Survival, Success

## Abstract

**Purpose:**

This review aimed to evaluate the existing evidence on the effects of dietary factors and nutrients on dental implant osseointegration.

**Methods:**

This scoping review was conducted in accordance with the Preferred Reporting Items for Systematic Reviews and Meta-Analyses Extension for Scoping Reviews (PRISMA-ScR) guidelines. Electronic searches were performed in PubMed, Web of Science, and the Cochrane Library, supplemented by manual searches of relevant journals. Human clinical studies published up to November 2025 were included.

**Results:**

Out of 592 screened studies, a total of 27 clinical studies were included, comprising 13 cohort studies, 8 randomized controlled trials, 3 cross-sectional studies, 2 retrospective case–control studies, and 1 uncontrolled interventional study. Most studies focused on vitamin D, whereas evidence for other micronutrients was limited. Fifteen studies reported a positive association between vitamin D and implant osseointegration. Five studies reported no significant association, while two studies investigating vitamin B and vitamin C showed that vitamin B did not influence postoperative pain or paresthesia and vitamin C improved soft tissue healing. Sufficient vitamin D status was associated with significantly higher implant stability quotient (ISQ) values, reduced early dental implant failure (EDIF), and lower marginal bone loss (MBL).

**Conclusion:**

Current evidence indicates that sufficient vitamin D status may support early osseointegration and implant stability, while deficiency is linked to less favorable early outcomes. Well-designed randomized controlled trials with standardized outcome measures, longer follow-up, and evaluation of additional micronutrients are needed.

## Background

Since Brånemark’s groundbreaking discovery of osseointegration in the 1960s, the field of dental implantology has seen extensive scientific progress and innovation [1]. Osseointegration, defined as the direct structural and functional connection between bone and the surface of the load bearing implant, is a prerequisite for long-term success of dental implants [2]. Several factors influence osseointegration such as local factors including surgical techniques, implant material, design, surface characteristics and patient related factors including general health of the patient, immune response, medications, radiation, bone volume and bone quality. Most research has been focusing on the surgical and the implant related factors over the last decades, with less emphasis on systemic factors [2, 3], where in particular the effects of smoking and uncontrolled diabetes have been extensively studied [4].

Recently, there has been an increasing interest in the role of micronutrients and nutraceuticals in physiological regulation processes in the body [5]. The skeletal system relies on several essential minerals and vitamins including calcium, fluoride, potassium, magnesium, vitamin B6, vitamin D and zinc for regular function. Rickets represents one of the most famous known disease based on deficiency of vitamin D. In line with this, a positive relationship between vitamin D levels and the success of dental implants has been reported [6]. Animal studies indicate that vitamin D deficiency can impair osseointegration and increase early dental implant failure. Supplementation of vitamin D is reported to enhance osseointegration in animals with systemic comorbidities such as vitamin D deficiency, diabetes mellitus, osteoporosis and chronic kidney disease [7]. However, due to the limited number of human studies investigating the effects of vitamin D supplementation on implant outcomes, the current evidence remains weak and preliminary [4, 8]. Moreover, nutrients such as calcium, phosphorus, magnesium, and zinc are also essential for bone formation and remodeling, while vitamin C contributes to collagen synthesis and soft-tissue healing around implants [9].

Despite the recent growing interest and research insights, there is limited evidence on how the deficiency or supplementation of other micronutrients (e.g., boron, selenium, copper, or iron) influences osseointegration or the long-term implant survival and success. Data regarding optimal dosing regimens, timing, and synergistic effects among nutrients are also lacking. A previous review explored the effects of dietary supplements and nutraceuticals on dental implant osseointegration; however, most of the included studies were preclinical animal experiments with limited evidence from human research [10].

This scoping review aims to systematically map and evaluate the existing clinical evidence on the relationship between dietary factors and nutrients and the outcomes of dental implant treatment including osseointegration, implant stability, marginal bone loss, peri-implant tissue health and implant survival/success, implant failure incidents. In particular, it seeks to address whether the deficiency of such nutrients may reduce implant survival and success. By identifying current knowledge gaps, this review will help guide future research and clinical recommendations on the role of nutrition in oral implantology.

## Materials and methods

### Protocol and registration

Our protocol was drafted using the Preferred Reporting for Systematic Reviews and Meta-analyses Extension for Scoping Reviews (PRISMA-ScR) model [11]. The protocol of the current study is registered in PROSPERO (International Prospective Register of Systematic Reviews) under CRD420251172055 (https://www.crd.york.ac.uk).

### PICO question

The clinical focus question was formulated by the PICO (Population, Intervention, Comparator, Outcome) framework as follows:

Population: Human patients receiving dental implant treatment regardless of age or sex, in whom systemic nutritional status, dietary intake, or supplementation was assessed.

Intervention: Dental implant installation in patients with defined dietary or micronutrient status.

Comparison: Dental implant placement in patients with different nutritional statuses.

Outcome: Implant-related clinical outcomes, including: implant stability quotient (ISQ), early dental implant failure (EDIF), implant survival / success, implant failure, marginal bone loss (MBL), and clinical parameters such as clinical attachment level, probing depth, bleeding index.

### Focus question

What are the short and long-term beneficial or detrimental effects of nutrition on dental implant success and survival?

### Literature search strategy

A comprehensive electronic and manual search was conducted in PubMed, Web of Science and Cochrane Library for the nutrients that impact bone metabolism using the following Medical Subjects Headings (MeSH) terms: “dental implants”, “osseointegration”, “implant survival”, “implant success”, “marginal bone loss”, “peri-implantitis”, “bone-to-implant-contact” together with nutrient terms such as “dietary supplement”, “micronutrients”, “nutraceutical”, “vitamin” (Table 1). Additionally, a hand-search were carried out in relevant oral implantology journals such as Clinical Oral Implant Research, International Journal of Implant Dentistry, International Journal of Oral Implantology, and Clinical Implant Dentistry and Related Research. Bibliographies of relevant publications were also cross searched.

**Table 1:** Search strategy

Databases	Search Algorithm	Results
PubMed (Medline)	(“Dental Implants“[Majr] OR “Osseointegration“[Majr] OR “Peri-Implantitis“[Majr] OR peri-implantitis[tiab] OR periimplantitis[tiab] OR “peri-implant mucositis“[tiab] OR “peri-implant disease*“[tiab] OR “dental implant*“[tiab] OR “implant survival“[tiab] OR “implant success“[tiab] OR “marginal bone loss“[tiab] OR “bone-to-implant contact“[tiab] OR “bone to implant contact“[tiab] ) AND ( nutraceutical*[tiab] OR micronutrient*[tiab] OR “dietary supplement*“[tiab] OR “vitamin D“[tiab] OR vitamin*[tiab] OR calcium[tiab] OR magnesium[tiab] OR fluoride[tiab] OR zinc[tiab] OR iron[tiab] OR copper[tiab] OR selenium[tiab] OR boron[tiab] OR resveratrol[tiab] OR diet*[tiab] OR nutrition*[tiab] ) NOT ( coating*[tiab] OR “surface modification“[tiab] OR surface*[tiab] OR anodiz*[tiab] OR sandblast*[tiab] OR etch*[tiab] OR SLA[tiab] OR SLActive[tiab] OR roughness[tiab] OR nanotub*[tiab] OR hydroxyapatite[tiab] OR “calcium phosphate“[tiab] OR CaP[tiab] OR “titanium nitride“[tiab] OR bioglass[tiab] OR “bioactive glass“[tiab] OR “bioactive coating*“[tiab] ) AND humans[MeSH Terms] AND english[lang]	364
Web of Science	TS=(( “dental implant*” OR osseointegration OR “peri-implantitis” OR periimplantitis OR “peri-implant mucositis” OR “peri-implant disease*” OR “implant survival” OR “implant success” OR “marginal bone loss” OR “bone-to-implant contact” OR “bone to implant contact” ) NEAR/10 ( nutraceutical* OR micronutrient* OR “dietary supplement*” OR vitamin* OR calcium OR magnesium OR fluoride OR zinc OR iron OR copper OR selenium OR boron OR resveratrol OR diet* OR nutrition* ))AND TS=(human* OR patient* OR clinical*) NOT TS=( coating* OR “surface modification” OR surface* OR anodiz* OR sandblast* OR etch* OR SLA OR SLActive OR roughness OR nanotub* OR hydroxyapatite OR “calcium phosphate” OR CaP OR “titanium nitride” OR bioglass OR “bioactive glass” OR “bioactive coating*”)	86
Cochrane Library	((dental implant OR osseointegration OR implant survival OR implant success OR marginal bone loss OR bone-to-implant contact) AND (nutraceutical OR micronutrient OR dietary supplement OR vitamin OR calcium OR magnesium OR fluoride OR zinc OR iron OR copper OR selenium OR boron OR resveratrol OR diet OR nutrition) AND (human OR patient OR clinical)) NOT (material OR biomaterial OR coating OR surface OR SLA OR SLActive OR roughness OR hydroxyapatite OR topical)	142

Using the search algorithm described above, a total of 592 studies were found. All studies were entered into EndNote (version 2025; Clarivate) to eliminate duplicates.

### Eligibility criteria

Human, clinical trials published in the medical literature up to November 2025 in English language were included. The detailed inclusion criteria comprised of human clinical studies investigating the effect of nutrients and dietary factors reporting osseointegration, implant survival/success, MBL, ISQ, and peri-implant tissue health; these studies had to include information on systemic nutrient status, intake, or supplementation. Exclusion criteria were case reports, technical reports, animal studies, in vitro studies, and review papers. Studies were excluded if they did not fit into the conceptual framework of the study, focused on topical application of micronutrients for example onto the implant surface such as vitamin D coated implants, magnesium/fluoride-coated surfaces, anodized/HA, drug-eluting implants.

### Quality assessment and risk of bias

Two authors (B.S-I. and V.H.) independently assessed the risk of bias for each study using domain-based evaluation tools. Randomized controlled trails (RCTs) were assessed using the revised Cochrane Risk of Bias tool (RoB 2) [12]. Five domains were assessed for RoB 2, including bias arising from the randomization process, deviations from intended interventions, missing outcome data, measurement of the outcome, and selection of the result. The risk bias was categorized as low risk, some concerns, or high risk of bias.

For non-randomized studies, the Risk Of Bias In Non-randomized Studies of Interventions (ROBINS-I) tool [13] was used across seven domains including bias due to confounding, selection of participants into the study, classification of interventions, deviations from intended interventions, missing data, measurement of outcomes, and selection of the reported result.

### Statistical analysis

Although this study was designed as a scoping review, a quantitative synthesis of findings was performed when sufficient methodological and outcome comparability were available for meaningful pooling of a data subset. Quantitative synthesis and forest plots were generated using RevMan Web. For continuous outcomes such as ISQ and MBL, pooled mean differences (MDs) with 95% confidence intervals (CIs) were calculated using the inverse-variance method with a random-effects model. For dichotomous outcomes (early dental implant failure), risk ratios (RRs) with 95% CIs were calculated using the Mantel–Haenszel method with a fixed-effect model. Heterogeneity was assessed using the I² statistic. Outcomes not suitable for meta-analysis were summarized qualitatively.

## Results

### Study selection

**Fig. 1:**
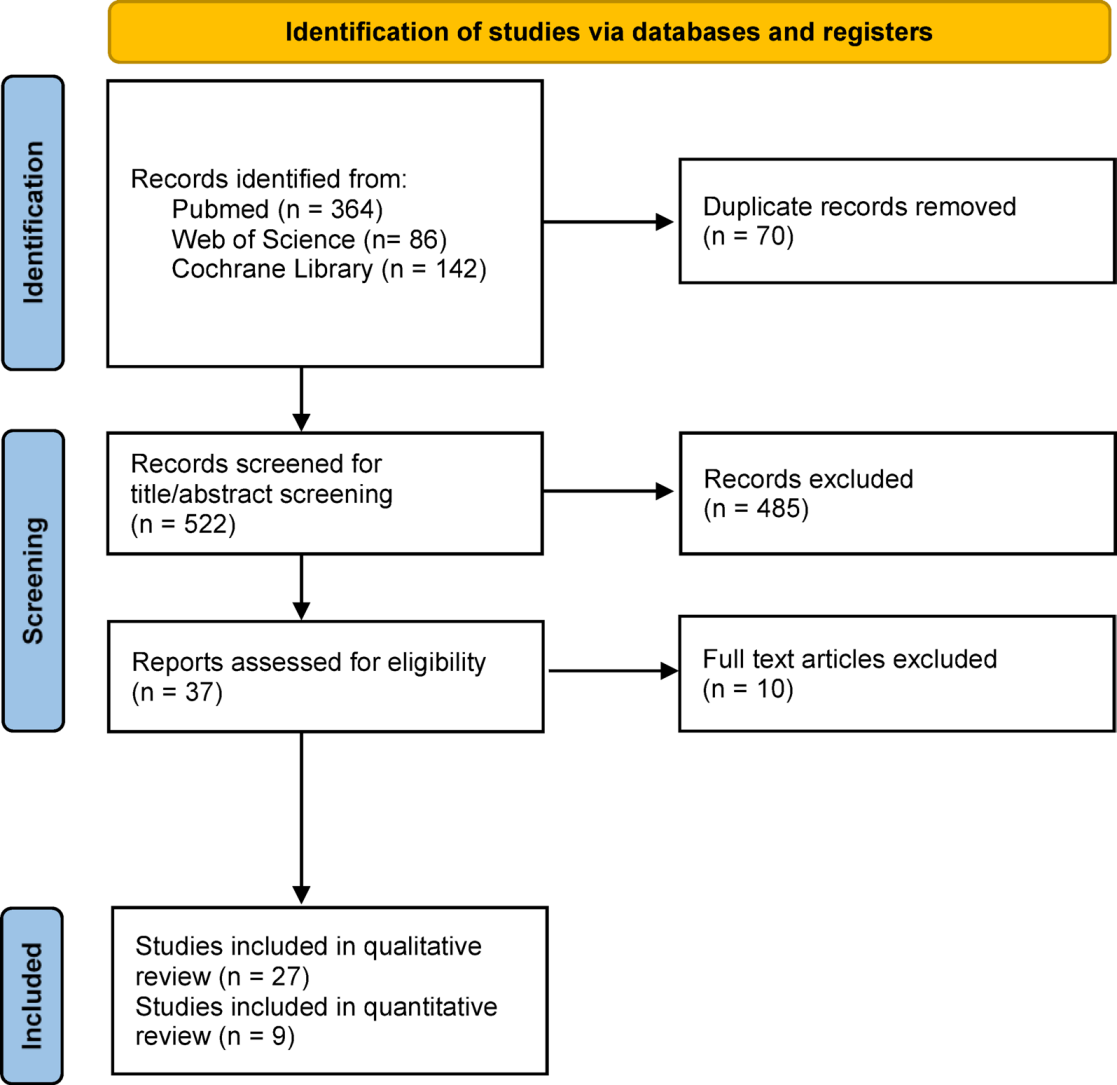
PRISMA flow chart of the review

A total of 522 studies were identified through the electronic search and after removal of duplicates. Following title and abstract screening, 37 articles were retained for full-text evaluation. Of these, 10 were excluded because they did not directly investigate the influence of nutrition on implant osseointegration and did not meet the inclusion criteria.　Eventually 27 articles were included in the systematic review (qualitative analysis), and 9 articles were included in the meta-analysis (quantitative analysis) (Fig. 1) (Tables 2, 3 and 4).

**Table 2:** Studies reporting the early outcomes of the effects of nutrition and implant osseointegration (outcomes assessed ≤ 12 months)

No.	References	Study design	Sample size	Measured parameters	Follow-up	Main findings
1	Grigoras et al. [21]	Prospective cohort study	166 patients; 556 implants	Short term implant survival	Up to 12 months	No statistically significant correlation between vitamin D levels and early implant failure.
2	Dulinska-Litewka et al. [45]	Retrospective cohort study	72 patients; 115 implants	Gingiva height and bone level changes	3–4 months	Vitamin D deficiency showed significantly higher levels of bone loss.
3	Toy et al. [24]	Case control study	42 patients, 84 implants	MBL, ISQ, ELISA	2 -3 months	Low level of vitamin D may impair bone hemostasis around dental implants by altering RANKL and IL-10 levels.
4	Singh et al. [14]	RCT	40 patients	ISQ and radiographic bone changes	3 months	Vitamin D supplementation led to higher stability, more bone regeneration, and less bone loss.
5	Toy et al. [23]	Prospective cohort study	129 patients; 357 implants	ISQ and RFA	3 months	High levels of vitamin D may promote dental implant osseointegration.
6	Iqbal et al. [15]	RCT	100 patients; 100 implants	ISQ	3 months	There was strong positive correlation between vitamin D level and ISQ value at 3 months.
7	Al-Quisi et al. [25]	Prospective cohort study	108 patients; 128 implants	Insertion torque value and grayscale value on CBCT	4 months	Severe vitamin D3 deficiency could be associated with early dental implant failure.
8	Francis et al. [26]	Prospective cohort study	109 patients; 174 implants	EDIF	3–6 months	No correlation between patients’ low serum vitamin D levels and early implant failure.
9	Mohsen et al. [27]	Prospective cohort study	53 patients; 143 implants	EDIF and ISQ	12 weeks	A higher incidence of EDIF was associated with lower vitamin D levels.
10	Wach et al. [28]	Retrospective cohort study	2196 implants	MBL	3 months	A decrease in vitamin D was correlated with higher marginal bone loss.
11	Ghasemi et al. [16]	RCT	46 patients	Postoperative pain intensity and rate of paresthesia	3 months	No significant difference between pain intensity.
12	Singh et al. [29]	Retrospective cohort study	30 patients	MBL	6 months	The success of the implant is significantly affected by vitamin D.
13	Bhandage et al. [30]	Prospective cohort study	20 patients	ISQ	3–6 months	Positive influence of vitamin D on stability of implant.
14	Tabrizi et al. [46]	Prospective cohort study	90 patients	MBL	1 year	Low serum level of vitamin D may be associated with increased MBL.
15	Kwiatek et al. [17]	RCT	122 patients; 122 implants	MBL	12 weeks	Vitamin D levels have a significant influence on the bone level at the implant site during osseointegration.
16	Garg et al. [18]	RCT	31 patients	MBL	6 months	Supplementation improved systemic effects on accelerating bone formation around dental implants.
17	Singh et al. [44]	Retrospective cohort study	90 patients	EDIF	5 months	Increased incidence of EDIF with lowering of serum vitamin D levels.
18	Piccolotto et al. [36]	Cross-sectional study	33 patients	Peri-implant clinical parameters	8-week supplementation follow-up	Vitamin D serum do not seem to affect peri-implant health.
19	Li et al. [47]	RCT	128 patients	Soft tissue healing and pain response scores were evaluated.	14 days	Vitamin C supplementation improves postoperative healing.
20	Mangano et al.[ ﻿^4^]	Retrospective cohort study	885 patients; 1,740 implants	EDIF	4 months	No statistically significant correlation between EDIF and vitamin D serum levels.
21	Schulze-Späte et al. [19]	RCT	20 patients	Histology and histomorphometry (peri-implant bone response)	6–8 months	Significant association between increased vitamin D levels and number of bone-resorbing osteoclasts around graft particles.
22	Mangano et al. [43]	Retrospective cohort study	822 patients; 1,625 implants	Implant failure rate	4 months	No link between low serum levels of vitamin D and an increased risk of early implant failure.

RCT: randomized controlled trial; MBL: marginal bone loss; EDIF: early dental implant failure; ISQ: implant stability quotient; RFA: resonance frequency analysis; CBCT: cone-beam computed tomography

**Table 3:** Studies reporting on the long-term outcomes of the effects of nutrition and implant osseointegration (outcomes assessed > 12 months)

No.	References	Study design	Sample size/	Measured parameters	Follow-up	Main findings
1	Diachkova et al. [20]	RCT	384 patients	ISQ and MBL, peri-implantitis rate	Up to 10 years	Peri-implantitis rate was significantly higher in patients with severe vitamin D deficiency.
2	Cheng et al. [33]	Retrospective case-control study	73 patients; 291 implants	MBL	Up to 19 years	Excess vitamin D levels were associated with adverse implant outcomes.
3	Paz et al. [34]	Prospective uncontrolled interventional study	20 patients; 20 implants	Implant success rate	1 year after implant placement	Vitamin D levels were normalized, and all implants were successful.
4	Ustaoglu et al. [37]	Cross-sectional study	156 patients; 196 implants.	Peri-implant clinical parameters	Post-loading (implants functioning ≥ 12 months); cross-sectional assessment	The peri-implantitis group had the lowest level of vitamin D.
5	Acipinar et al. [38]	Cross-sectional observational study	53 patients; 90 implants	Peri-implant clinical parameters recorded.	Post-loading (implants functioning ≥ 12 months); cross-sectional assessment	FGF-23 and vitamin D seems to affect peri-implant bone health, and further studies are needed.

RCT: randomized controlled trial; MBL: marginal bone loss; EDIF: early dental implant failure; ISQ: implant stability quotient; FGF-23: Fibroblast growth factor-23; ELISA: enzyme-linked immunosorbent assay

**Table 4:** Qualitative summary of evidence for the effects of micronutrients on dental implant outcomes

Outcome category	No. of studies	Study designs	Risk-of-bias profile (ROB 2 / ROBINS-I)	Strength of evidence
Implant stability (ISQ)	7	RCT, cohort	Low (1), Moderate (5), Serious (1)	++
EDIF	5	Prospective, retrospective	Moderate (2), Serious (3)	+
Short-term MBL	7	Case control, prospective, retrospective, RCT	Moderate (2), Some concern (1), High (1), Serious (3)	++
Bone density / quality	2	RCT, prospective	Moderate (1), serious (1)	+
Soft tissue outcomes	5	RCT, retrospective, cross-sectional	Serious (1), High (1) Not assessed (3 cross-sectional)	+ / ++
Long-term outcomes	5	RCT, cohort, cross-sectional	Low (1), Serious (1), Critical (1), Not assessed (2 cross-sectional)	+

RCT: randomized controlled trial; EDIF: early dental implant failure; ISQ: implant stability quotient

### Primary characteristics

Of the 27 included articles, 13 were cohort studies (8 prospective and 5 retrospective), 8 were randomized controlled trials, 3 were cross-sectional studies, 2 were retrospective case–control studies, and 1 was an uncontrolled interventional study. Twenty-four studies investigated the role of vitamin D, one study examined the effects of vitamin B on pain and sensory problems related to inferior alveolar nerve damage, and one study focused on the effects of vitamin C supplementation in wound healing following dental implant placement.

### Risk of bias assessment

**Fig. 2:**
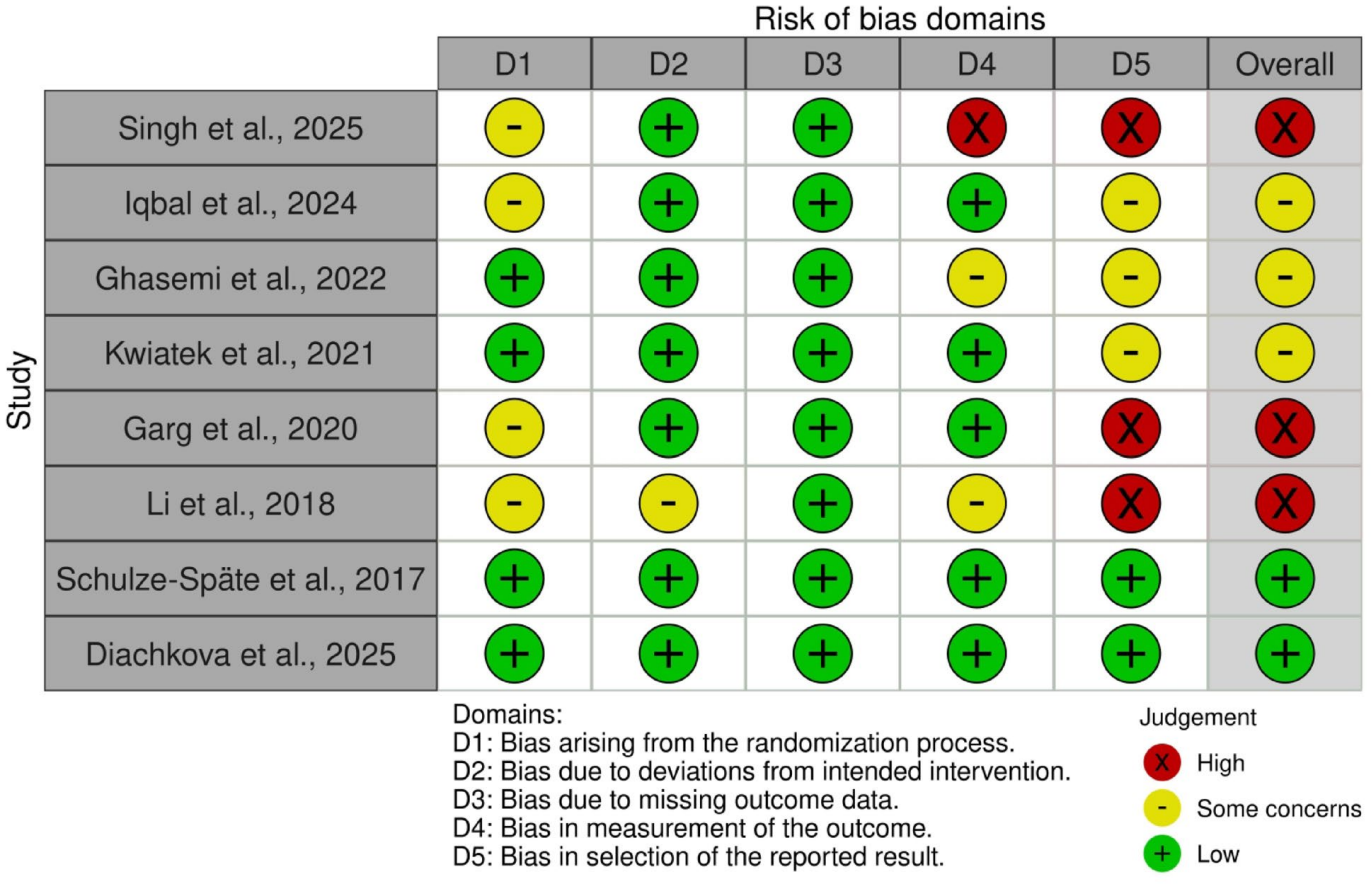
Risk of bias (RoB 2.0) assessment of the included randomized controlled trials

The basic characteristics of the included studies were shown in Table 4. Among the 8 included RCTs [9, 14–20], two studies were judged to be at “low risk” [19, 20], three studies were assessed as “some concerns” [15–17], and three studies were rated as “high risk” [9, 14, 18]. Four studies were judged to be “low risk” for the randomization process, employing a clearly described physical or block randomization with balanced group allocation [16, 17, 19, 20]. Most studies were judged to be at “low risk” to deviations from intended interventions (D2), and all trials were assessed as “low risk” due to missing outcome data (D3). In the domain of bias in measurement of the outcome (D4), several studies were judged as having “some concerns” or “high risk”, mainly due to limited reporting of outcome assessor blinding or the use of subjective outcome measures. For bias in selection of the reported result (D5), only two studies showed clear consistency between pre-specified and reported outcomes [19, 20] (Fig. 2).

**Fig. 3:**
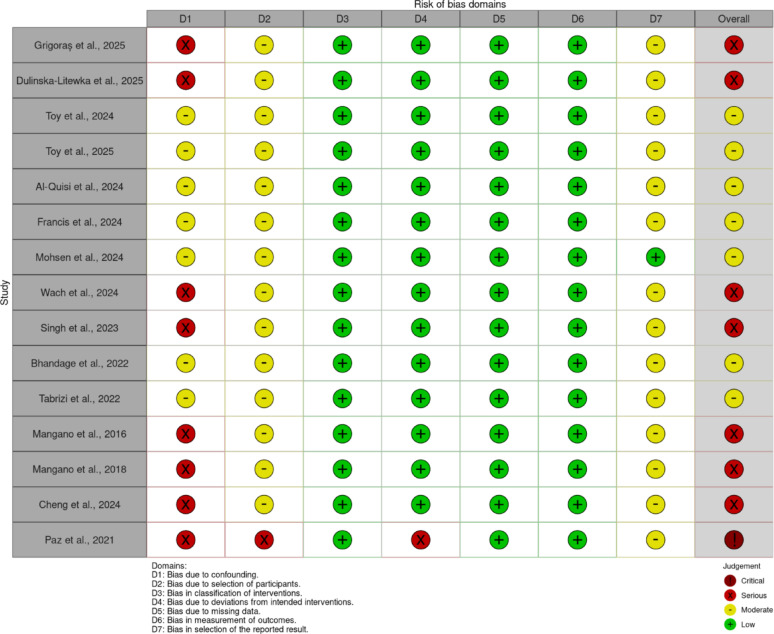
Risk of bias in non-randomized studies of interventions (ROBINS-I) assessment of the included studies

For the included non-randomized interventional and observational studies, most studies were rated as moderate to serious [4, 21–33] (Fig. 3). 1 study was deemed as critical due to the absence of a control group, inability to control for confounding, and selective outcome reporting [34].

### Osseointegration outcomes

The reported outcomes were categorized as early or late according to the different healing phases of dental implant treatment. Early outcomes included parameters related to early osseointegration and implant stability, such as ISQ, short-term marginal bone loss changes, early implant failure, bone density and quality outcomes and peri-implant soft tissue quality parameters [35]. Late outcomes reflected long-term peri-implant health and functional outcomes, including the incidence of peri-implant mucositis, peri-implantitis, long-term marginal bone loss, and implant survival [35].

#### Early outcomes

##### Implant stability

Implant stability was the most frequently reported outcome and was evaluated in seven studies using resonance frequency analysis (RFA). Of these, five studies (71%) demonstrated a statistically significant positive association between vitamin D status and implant stability, whereas two studies reported no significant relationship.

Toy et al. reported a postoperative ISQ value of 82.02 ± 4.98 in the vitamin D sufficient group versus 79.86 ± 5.86 in the insufficient group, which did not reach statistical significance (*p* > 0.05) [24]. Similarly, Diachkova et al. compared patients who received implant placement after normalization of serum vitamin D levels with those who underwent implantation during vitamin D supplementation and found no significant differences in implant stability. ISQ values at implant insertion ranged from 82 to 86 (group 1: 84 ± 1.4; group 2: 83.8 ± 1.4; *p* > 0.05), and at prosthetic insertion from 86 to 90 (group 1: 87.8 ± 1.4; group 2: 87.5 ± 1.4; *p* > 0.05) [20].

In contrast, the remaining five studies investigating implant stability reported a significant positive association with vitamin D levels [14, 15, 23, 27, 30]. Notably, Bhandage et al. demonstrated that for every 1 ng/mL increase in serum vitamin D concentration, the ISQ value increased significantly by 0.48 units at 3 months and 0.62 units at 6 months (*p* = 0.01 and *p* = 0.002, respectively) [30].

**Fig. 4:**
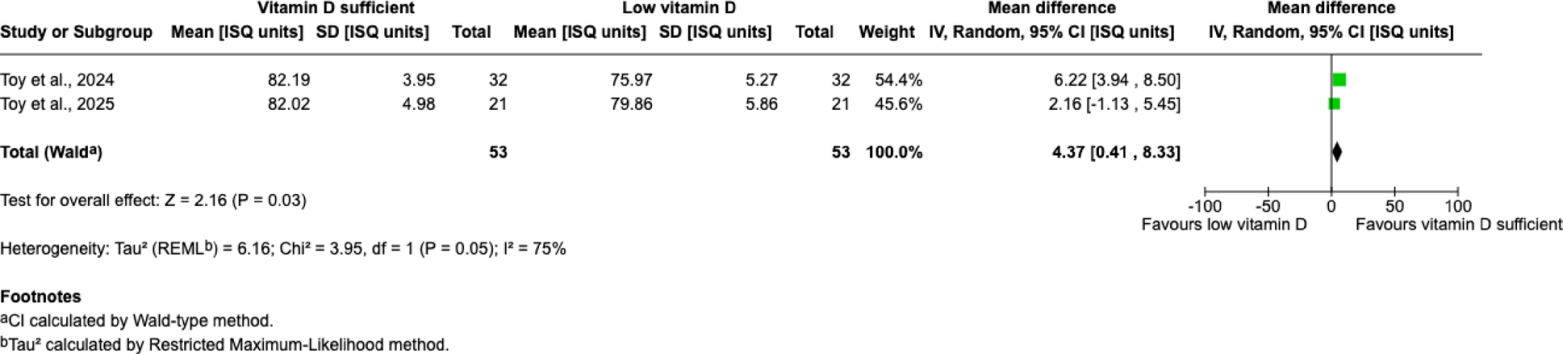
Forest plot comparing ISQ between vitamin D sufficient and low vitamin D groups at 3 months follow-up

Due to the heterogeneity in study design, only two studies with comparable methodology and follow-up duration were eligible for quantitative synthesis. Meta-analysis of these two studies showed a significantly higher ISQ at 3 months in vitamin D–sufficient patients compared with vitamin D–insufficient patients (mean difference = 4.37 ISQ units; 95% CI: 0.41–8.33; *p* = 0.03), although the heterogeneity remained substantial (I² = 75%) (Fig. 4).

##### Early dental implant failure (EDIF)

Out of 5 studies that reported EDIF, one study reported a statistically significant association between vitamin D deficiency and an increased EDIF [27], while four studies that observed higher EDIF rates in patients with low vitamin D levels did not reach a statistical significance [4, 21, 25, 32].

**Fig. 5:**
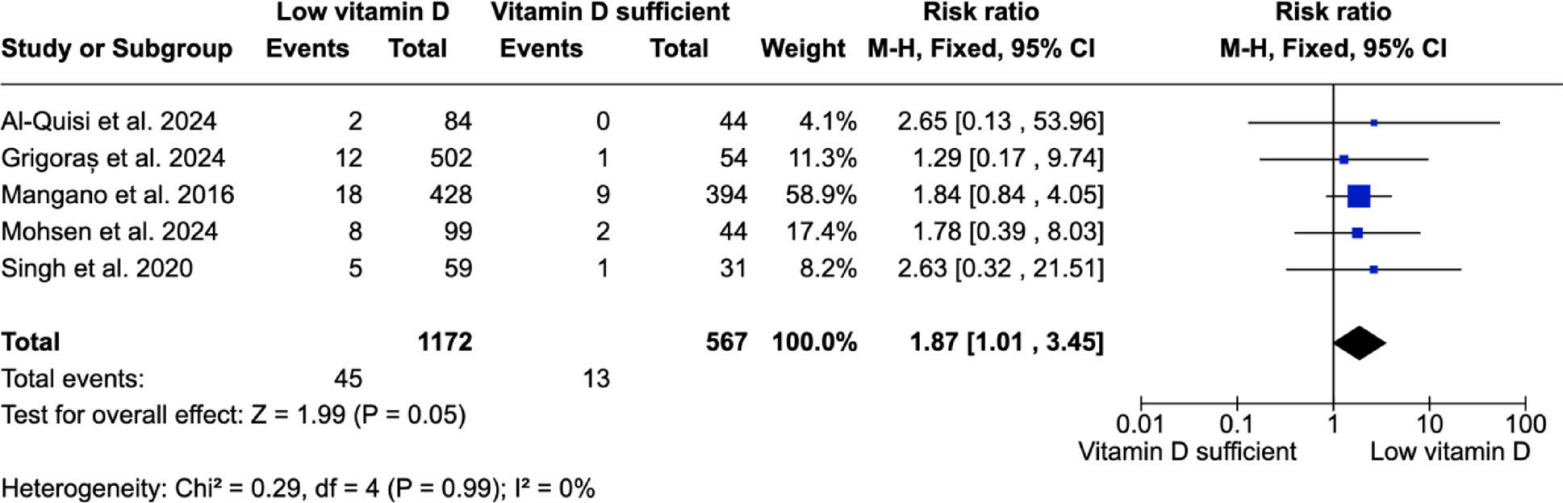
Forest plot comparing EDIF between vitamin D sufficient and low vitamin D groups

All five studies reporting EDIF were included in the quantitative synthesis. Pooled analysis demonstrated a significantly higher risk of EDIF in patients with low vitamin D levels compared with vitamin D sufficient patients (risk ratio = 1.87; 95% CI: 1.01–3.45; *p* = 0.05) (Fig. 5).

##### Short-term marginal bone loss (MBL) changes

**Fig. 6:**
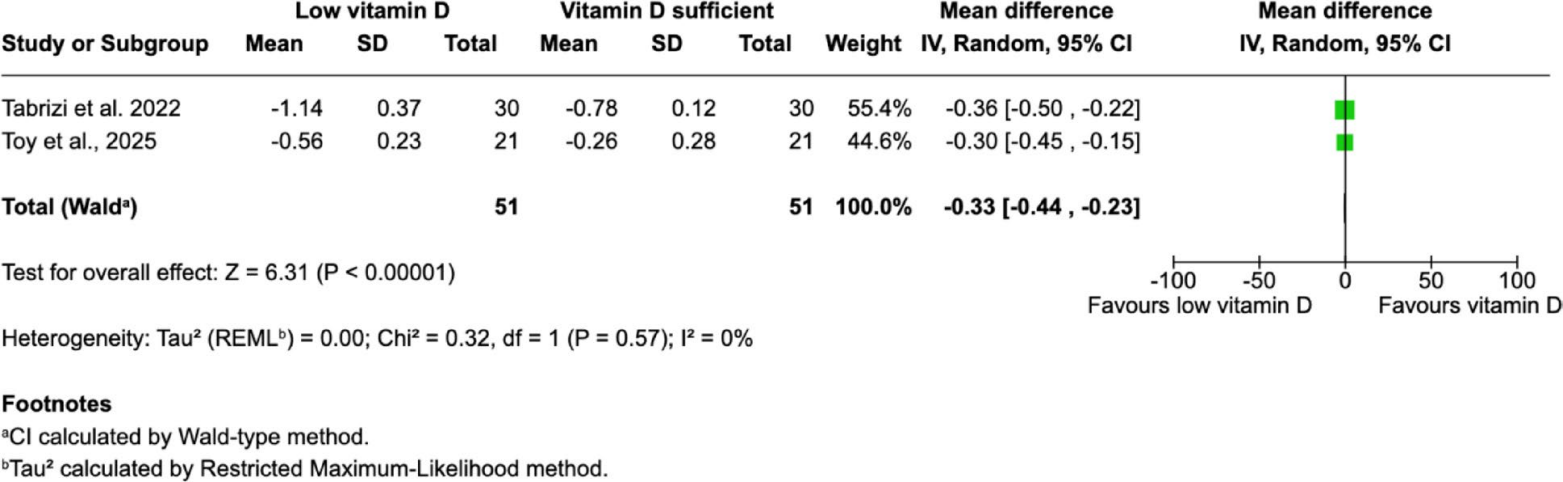
Forest plot comparing early MBL changes (≤ 12 months) between vitamin D sufficient and low vitamin D groups

Short-term MBL changes were evaluated in 7 studies during the early healing phase, reflecting early peri-implant bone remodeling and initial osseointegration. In studies where comparable data were available, quantitative synthesis demonstrated significantly greater early MBL in patients with low serum vitamin D levels compared with sufficient patients (Fig. 6) [24, 31]. Overall, most studies consistently reported increased MBL associated with low vitamin D [14, 17, 18, 24, 28, 29, 31].

##### Bone density and quality

A total of two studies reported on the bone density and quality outcomes in relation to vitamin D levels. In a study by Al-Quisi et al., in which most patients presented with deficient serum vitamin D3 levels (81 of 108 patients), a weak positive correlation was observed between serum vitamin D3 levels and alveolar bone density; however, severe vitamin D3 deficiency was associated with early implant failure despite favorable bone density and primary stability [25]. In another study, Schulze-Späte et al. used histological and histomorphometric analyses of bone core biopsies and reported that vitamin D supplementation was associated with increased cellular bone remodeling activity, reflected by higher osteoclast numbers [19].

##### Effects of micronutrients on peri-implant soft-tissue

A total of 5 studies reported on the effects of micronutrients including vitamin C and D on peri-implant soft tissue outcomes using clinical parameters, gingival phenotype measurements, peri-implant cervical fluid biomarkers and wound healing indices [9, 22, 24, 36, 37].

Among the four studies investigating vitamin D, three reported significant associations between low serum vitamin D levels and less favorable peri-implant soft tissue conditions. Dulinska et al. showed that vitamin D–deficient patients with thin gingival tissue (< 3 mm) exhibited significantly greater peri-implant bone loss at implant exposure (*p* = 0.040) and crown placement (*p* < 0.001) [22]. Similarly, Ustaoglu et al. found significantly lower serum vitamin D levels in patients with peri-implantitis compared with peri-implant healthy individuals (*p* < 0.05) [37]. At the molecular level, Toy et al. reported significantly higher peri-implant crevicular fluid RANKL levels and lower IL-10 levels in vitamin D insufficient patients compared with sufficient controls (*p* < 0.05) [24]. In contrast, one study by Piccolotto et al. found no significant association between serum vitamin D levels and peri-implant clinical soft tissue parameters (*p* > 0.05) [36].

Finally, one study evaluating vitamin C supplementation reported significantly improved early postoperative wound healing at 7–14 days compared with controls (*p* < 0.05) [9].

#### Long-term outcomes

Long-term outcomes assessing the effects of vitamin D on implant success and survival, incidents of peri-implantitis were reported in five studies with follow-up periods ranging from 1 to 19 years [20, 33, 34, 37, 38].

Diachkova et al., reported that all cases of peri-implantitis occurred in patients with vitamin D deficiency or severe deficiency (*p* = 0.04; *p* < 0.05) at 10-year follow-up [20]. Cheng et al., with the follow-up of 19 years, observed that vitamin D hypervitaminosis were associated with increased peri-implant bone loss and implant failure, particularly in the maxilla, suggesting a potential detrimental effect of excessive vitamin D levels over the long term [33].

### Summary of key findings

Overall, the available evidence suggests a positive relationship between sufficient vitamin D status and early dental implant osseointegration.

For marginal bone changes and peri-implant soft tissue outcomes, the findings were consistent but statistically heterogeneous, with several studies indicating greater bone loss, thinner gingival phenotypes, or a more pro-inflammatory peri-implant environment in the presence of vitamin D deficiency. Long-term outcomes were supported by limited and heterogeneous evidence, suggesting that both vitamin D deficiency and excess may be associated with unfavorable peri-implant conditions over time.

## Discussion

To the best of the authors knowledge, this scoping review is the first to comprehensively synthesize evidence from clinical human studies assessing the influence of nutrients on dental implant osseointegration. In recent years, there has been growing interest in investigating the effects of nutritional factors on osseointegration [1, 5, 6, 10, 39–41]. Both macronutrients and micronutrients may modulate bone metabolism and immune function, which are central to early implant healing and long-term peri-implant stability [42]. Among micronutrients listed in the EU Register of nutrition and health claims made on foods, several have been implicated in bone metabolism, including calcium, fluoride, magnesium, potassium, sodium, zinc, resveratrol, vitamin D, vitamin C, vitamin K, vitamin A, vitamin E, and vitamin B [10]. However, most clinical evidence in implant dentistry has focused on vitamin D, whereas evidence for other micronutrients remains limited. Notably, only two clinical studies in the present review evaluated micronutrients other than vitamin D (e.g. vitamins B and C), addressing postoperative pain-related outcomes and early soft-tissue healing, underscoring a substantial gap in clinical research [9, 16].

In this review, outcomes were interpreted according to the different healing phases of implant therapy. Early outcomes included parameters related to early osseointegration and implant stability, whereas late outcomes reflected longer-term peri-implant health and function, including incidence of peri-implant mucositis, peri-implantitis, long-term MBL and implant survival. Importantly, the current evidence remains highly heterogeneous, with substantial variability in study design, baseline nutritional assessment, supplementation protocols, outcome definitions, and follow-up intervals. Therefore, quantitative analysis was performed only when outcomes were comparable across studies in a specified data subset, while the remaining evidence in the broader thematic field evaluated in this study was synthesized qualitatively using a structured approach. The pooled analyses should be interpreted with caution due to the limited number of studies included and the heterogeneity in the respective study designs as well as outcome reporting. This highlights the need for standardized reporting and outcome measures in future clinical research addressing the effect of dietary factors and nutrients on dental implant success and survival.

Despite a growing number of studies examining vitamin D, the evidence base is dominated by observational designs, while randomized controlled trials remain scarce. In addition, follow-up periods are predominantly short-term, limiting conclusions regarding sustained implant performance. Long-term outcomes were reported in only a small number of studies, with follow-up extending up to 10 and 19 years in individual reports [20, 33]. The short-term outcomes investigating the effects of vitamin D on dental implants show that the deficiency of vitamin D effected the early healing phase, with an increased EDIF compared to the sufficient vitamin D group [21, 25, 27, 29, 43, 44]. Conversely, data also suggest that excessively high serum vitamin D levels may be associated with adverse implant outcomes, including increased implant failure and peri-implant bone loss [33]. Taken together, the findings are compatible with the possibility that both deficiency and excess vitamin D could be unfavorable, but the current evidence is insufficient to define an optimal therapeutic range for implant patients.

In the included literature, serum 25-hydroxyvitamin D levels were classified as deficiency or insufficiency using a threshold of < 30 ng/mL, while a subset of studies further categorized insufficiency as 20–29 ng/mL, deficiency as < 20 ng/mL, and severe deficiency as < 10 ng/mL. Seven clinical studies reported a defined vitamin D supplementation regimen for deficient or insufficient individuals. These included monthly high-dose cholecalciferol administration of 60,000 IU for at least three months, daily moderate-dose protocols of 5,000–8,000 IU/day administered for approximately 12 weeks, weekly high-dose regimens of 50,000 IU/week for eight weeks, a structured pre-surgical supplementation program providing 6,000 IU/day for six weeks, and an endocrinologist-guided individualized correction approach followed by maintenance dosing of 1,000–2,000 IU/day after achieving target serum levels [15, 17–20, 34, 36]. Notably, although multiple co-factors such as vitamin K, magnesium, boron, manganese and other micronutrients have been shown to enhance the absorption and metabolism of vitamin D supplements [10, 39], the included studies did not systematically assess these additional micronutrient co-factors in relation to implant-related outcomes.

Based on the available clinical evidence, the following clinical recommendations are considered: vitamin D screening may be considered in patients planning to undergo implant therapy, particularly in individuals at higher risk of deficiency (limited sun exposure, older age, malabsorption, osteoporosis, chronic illness, or a history of early implant failure), as baseline assessment may support individualized pre-operative optimization. Vitamin D deficiency may represent a potentially modifiable risk factor in the early osseointegration, since pooled evidence suggested a higher risk of early dental implant failure and several studies reported less favorable early marginal bone changes in deficient patients. Furthermore, the available evidence remains heterogeneous and does not support a standardized dosing regimen; therefore, supplementation should follow established medical deficiency guidelines. Importantly, both deficiency and excessive serum vitamin D levels should be avoided, given limited long-term data suggesting potentially unfavorable outcomes at both extremes.

## Conclusion

Based on the available clinical evidence, sufficient vitamin D status appears to support early dental implant osseointegration and stability, whereas deficiency is associated with less favorable early outcomes. Future research should focus on randomized controlled trials with standardized outcome measures, uniform timing of outcome assessment, and with adjustment for key confounding factors, such as smoking status, systemic comorbidities, bone quality, and implant and site-related variables. In addition, future studies should consider the potential influence of other micronutrients known to affect vitamin D absorption and metabolism. Longer follow-up periods are needed to determine whether optimization of vitamin D or other micronutrients status results in clinically meaningful improvements in implant survival and peri-implant health.

## Data Availability

The datasets generated during and/or analyzed by the authors during this study are available from the corresponding author upon request.

## Abbreviations

PRISMA-ScR: Preferred Reporting for Systematic Reviews and Meta-analyses Extension for Scoping Reviews
PICO: Population, Intervention, Comparator, Outcome
ISQ: Implant stability quotient
RFA: Resonance frequency analysis
EDIF: Early dental implant failure
MBL: Marginal bone loss
RCT: Randomized controlled trial
